# An Outer Membrane Receptor of *Neisseria meningitidis* Involved in Zinc Acquisition with Vaccine Potential

**DOI:** 10.1371/journal.ppat.1000969

**Published:** 2010-07-01

**Authors:** Michiel Stork, Martine P. Bos, Ilse Jongerius, Natasja de Kok, Ingrid Schilders, Vincent E. Weynants, Jan T. Poolman, Jan Tommassen

**Affiliations:** 1 Department of Molecular Microbiology, Utrecht University, Utrecht, The Netherlands; 2 GlaxoSmithKline Biologicals, Rixensart, Belgium; Northwestern University Feinberg School of Medicine, United States of America

## Abstract

Since the concentration of free iron in the human host is low, efficient iron-acquisition mechanisms constitute important virulence factors for pathogenic bacteria. In Gram-negative bacteria, TonB-dependent outer membrane receptors are implicated in iron acquisition. It is far less clear how other metals that are also scarce in the human host are transported across the bacterial outer membrane. With the aim of identifying novel vaccine candidates, we characterized in this study a hitherto unknown receptor in *Neisseria meningitidis*. We demonstrate that this receptor, designated ZnuD, is produced under zinc limitation and that it is involved in the uptake of zinc. Upon immunization of mice, it was capable of inducing bactericidal antibodies and we could detect ZnuD-specific antibodies in human convalescent patient sera. ZnuD is highly conserved among *N. meningitidis* isolates and homologues of the protein are found in many other Gram-negative pathogens, particularly in those residing in the respiratory tract. We conclude that ZnuD constitutes a promising candidate for the development of a vaccine against meningococcal disease for which no effective universal vaccine is available. Furthermore, the results suggest that receptor-mediated zinc uptake represents a novel virulence mechanism that is particularly important for bacterial survival in the respiratory tract.

## Author Summary

The outer membrane protects Gram-negative bacteria against harmful compounds from the environment. Nutrients usually pass this barrier by passive diffusion via pore-forming proteins. However, nutrients that are scarce in the environment are taken up via an active, receptor-mediated process. The vast majority of Gram-negative bacterial receptors described to date are involved in iron acquisition. Since free iron is scarce in the human host, these receptors constitute important virulence factors. In a search for putative vaccine components, we have characterized here a new receptor of *Neisseria meningitidis*, a resident of the nasopharynx that occasionally causes sepsis and meningitis. We show that expression of this receptor is induced under zinc limitation and that the protein is involved in the uptake of zinc. Homologues of this protein are present in many other Gram-negative pathogens, particularly in those residing in the respiratory tract, suggesting that receptor-mediated zinc acquisition is important for bacteria residing in this niche. We also found that the protein is highly conserved among *N. meningitidis* isolates and that it induces bactericidal antibodies upon immunization of mice. Therefore, the protein appears an excellent candidate for the development of a vaccine against *N. meningitidis*, for which no universal vaccine is available yet.

## Introduction

The cell envelope of Gram-negative bacteria consists of two membranes, the inner and the outer membrane, which are separated by the periplasm containing the peptidoglycan layer. The outer membrane forms a barrier for harmful compounds from the environment. Most nutrients can pass the outer membrane by passive diffusion via abundant channel-forming outer membrane proteins, collectively called porins. However, diffusion is not an option when the extracellular concentration of a nutrient is low. This is usually the case, for example, with iron. Pathogens are confronted with low concentrations of free iron within the human host, where iron is bound by iron-transport and -storage proteins, such as lactoferrin and transferrin. Hence, efficient iron acquisition mechanisms constitute important virulence factors and have been studied extensively in many pathogens [1], [2].

When grown under iron-limiting conditions, Gram-negative bacteria induce the synthesis of outer membrane proteins that function as receptors for the iron-binding proteins of the host, for heme, or for siderophores, which are small iron-chelating compounds produced and secreted by the bacteria under iron limitation. The resolved crystal structures of such receptors revealed 22-stranded β-barrels, which do not form open channels but are closed by an N-terminal plug domain [3]. After binding of the ligand to the receptor, the subsequent uptake of the nutrient is an active process that requires the energy of the proton gradient across the inner membrane, which is coupled to the receptors in the outer membrane via a complex of three proteins, the TonB complex [4], [5].

While iron-acquisition mechanisms have been studied extensively in many Gram-negative bacteria, little is known yet about the transport of other essential heavy metals, such as zinc and manganese, across the bacterial outer membrane. The concentrations of also these trace elements are low in the human host, which responds to infections, amongst others, by the production of metallothioneins and calprotectin, thereby reducing the availability of metals to the invading pathogens [6], [7]. Therefore, Gram-negative pathogens likely possess effective mechanisms for the acquisition of these metals, which may or may not resemble the iron-acquisition systems.

*Neisseria meningitidis* is an obligate human pathogen that can colonize the nasopharyngeal mucosa asymptomatically. Occasionally the bacterium enters the bloodstream and can cause sepsis and meningitis with a high mortality rate [8]. While vaccines based on the capsular polysaccharides are available for most pathogenic serogroups of *N. meningitidis*, a vaccine against serogroup B meningococci is lacking. The polysaccharide capsule of the serogroup B strains is poorly immunogenic due to its resemblance to human glycoproteins [9]. Thus, subcapsular antigens are being studied as alternative vaccine components; however, these studies are frustrated by the high antigenic variability of the major outer membrane proteins. Therefore, attention has shifted to minor antigens, including the TonB-dependent receptors.

When grown under iron limitation, *N. meningitidis* produces TonB-dependent receptors for lactoferrin [10], transferrin [11], hemoglobin [12], [13] and enterobactin [14], all involved in the uptake of iron. Based on homology searches, Turner *et al.* identified seven additional genes for putative TonB-dependent family (Tdf) members in the available genome sequences of three Neisserial strains [15]. Interestingly, the expression of some of these *tdf* genes appeared unaffected by iron availability in various microarray studies [16], [17], indicating that their products might be implicated in the transport of metals other than iron. Here we studied the regulation of the synthesis, the function, and the vaccine potential of one of these receptors and show that this receptor is involved in the uptake of zinc. We therefore named this protein, encoded by locus NMB0964 in the genome sequence of strain MC58 [18], ZnuD for zinc uptake component D.

## Results

### Regulation of *znuD* expression by zinc

**Figure 1:**
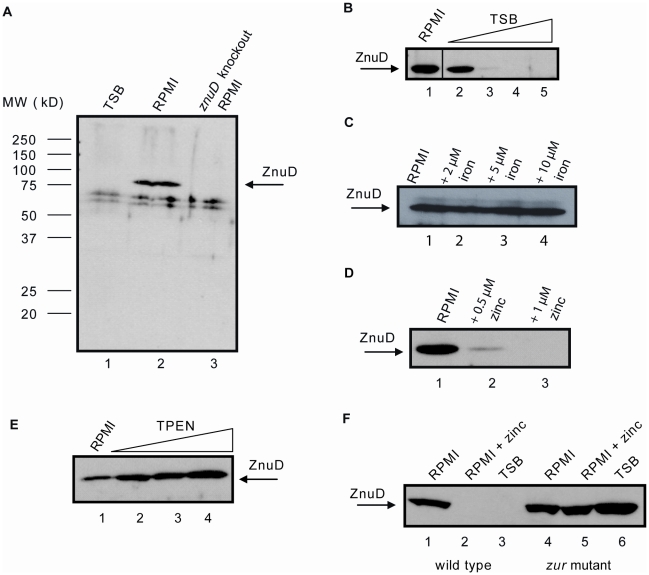
Regulation of *znuD* gene expression. Western blots of cell lysates using rabbit antiserum against ZnuD. (A) HB-1 grown in TSB (lane 1), RPMI (lane 2) and the *znuD* knockout strain grown in RPMI (lane 3). (B) HB-1 grown in RPMI supplemented with 0, 2, 4, 6 and 8% TSB (lanes 1–5, respectively). (C) HB-1 grown in RPMI supplemented with 0, 2, 5, and 10 µM FeCl_3_ (lanes 1–4, respectively). (D) HB-1 grown in RPMI supplemented with 0, 0.5 or 1 µM ZnSO_4_ (lanes 1–3, respectively). (E) HB-1 grown in RPMI supplemented with 0, 0.1, 0.5 or 1 µM TPEN (lanes 1–4, respectively). (F) HB-1 (lanes 1–3) and the *zur* mutant (lanes 4–6) grown in RPMI (lanes 1 and 4), RPMI with 0.6 µM ZnSO_4_ (lanes 2 and 5) or TSB (lanes 3 and 6).

To study the expression of *znuD* in *N. meningitidis*, we raised a polyclonal antiserum against the protein produced in *Escherichia coli* in inclusion bodies. While the antiserum did recognize the protein produced in *E. coli*, we could never detect ZnuD when whole cell lysates of *N. meningitidis* strain HB-1, an unencapsulated derivate of serogroup B strain H44/76, were analyzed on Western blots after growth in tryptic soy broth (TSB) (Figure 1A, lane 1). However, when the bacteria were grown in chemically defined RPMI medium, ZnuD was detectable in the lysates (Figure 1A, lane 2). The specificity of the signal detected was demonstrated by its absence in a constructed *znuD* knockout strain (Figure 1A, lane 3). We noticed that the addition of even small amounts of TSB to RPMI negatively affected ZnuD synthesis (Figure 1B), suggesting that TSB contains a compound that represses the transcription of *znuD*. RPMI does not contain a source of trace metals. Since Tdf members are usually regulated by iron availability, we first tested whether *znuD* expression could be repressed by adding an iron source; however, addition of even up to 10 µM FeCl_3_ to the medium did not affect ZnuD production (Figure 1C). Next, we decided to test whether a cocktail of trace metals, consisting of 340 nM ZnSO_4_, 160 nM Na_2_MoO_4_, 800 nM MnCl_2_, 80 nM CoCl_2_ and 80 nM CuSO_4_ (final concentrations), could repress *znuD* expression, which indeed appeared to be the case. Then, all these metal salts were tested separately, and specifically zinc, even at sub-µM concentrations, appeared to repress *znuD* expression (Figure 1D). Since standard RPMI is not supplemented with a specific zinc source, the available zinc required for bacterial growth is presumably derived from the water or the salts used to constitute the medium. The zinc concentration in the standard RPMI medium measured by inductively coupled plasma mass spectrometry (ICP-MS) was found to be ∼110 parts per billion (∼1.69 µM), which is apparently sufficient for growth of the bacteria but insufficient for repression of *znuD* expression.

The zinc regulation of *znuD* expression was further evaluated by supplementing the RPMI medium with the specific zinc chelator N,N,N′,N′-Tetrakis-(2-pyridylmethyl)-ethylenediamine (TPEN), which resulted in a dose-dependent increase in ZnuD synthesis (Figure 1E). Concentrations of TPEN above 1 µM totally inhibited bacterial growth presumably due to total depletion of zinc from the medium. The growth defect induced by TPEN could be restored by the addition of zinc (not shown). The zinc-dependent regulation of *znuD* expression was further confirmed by real-time quantitative PCR (RT-qPCR) using total RNA obtained from cultures grown in RPMI supplemented or not with either 0.5 µM ZnSO_4_ or 0.5 µM TPEN. The data showed a 13.8±1.3-fold repression in the presence of zinc and a 3.8±1.2-fold induction in the presence of TPEN. The fold difference between added TPEN and zinc was 52.6.

### Role of the transcriptional regulator Zur in *znuD* expression

In *E. coli*, the zinc uptake regulator Zur has been shown to regulate the expression of the *znuACB* operon. The genes of this operon encode the periplasmic substrate-binding protein, the ATPase and the integral inner membrane component, respectively, of an ABC transporter required for the transport of zinc from the periplasm into the cytoplasm [19]. In the presence of zinc, Zur binds a Zur-binding element (consensus sequence GAAATGTTATANTATAACATTTC) in the promoter of the *znuACB* operon and thereby blocks transcription [20].

In the genome sequence of *N. meningitidis* strain MC58, we identified homologues of the *E. coli zur* gene, *i.e.* NMB1266, and of a putative *znuCBA* operon, *i.e.* NMB0588, NMB0587, and NMB0586. In addition, we found sequences resembling the *E. coli* Zur binding sequence in the regions upstream of the *znuD* (GtAATGTTATATaATAACAaact) and *znuC* (cAAAcGTTATACagTAtCATaTC) (identical nucleotides to the *E. coli* consensus are in capital case). To confirm the involvement of Zur in the regulation of *znuD* expression, we generated a *zur* mutant of strain HB-1, which, indeed, produced ZnuD constitutively (Figure 1F). Also, RT-qPCR demonstrated the involvement of Zur in the expression of *znuA* and *znuD*, as *znuA* and *znuD* expression levels increased 5.0±0.8-fold and 34.0±0.8-fold, respectively, in the *zur* mutant compared to its parent strain both grown in RPMI supplemented with 0.5 µM ZnSO_4_.

### ZnuD facilitates zinc acquisition

**Figure 2:**
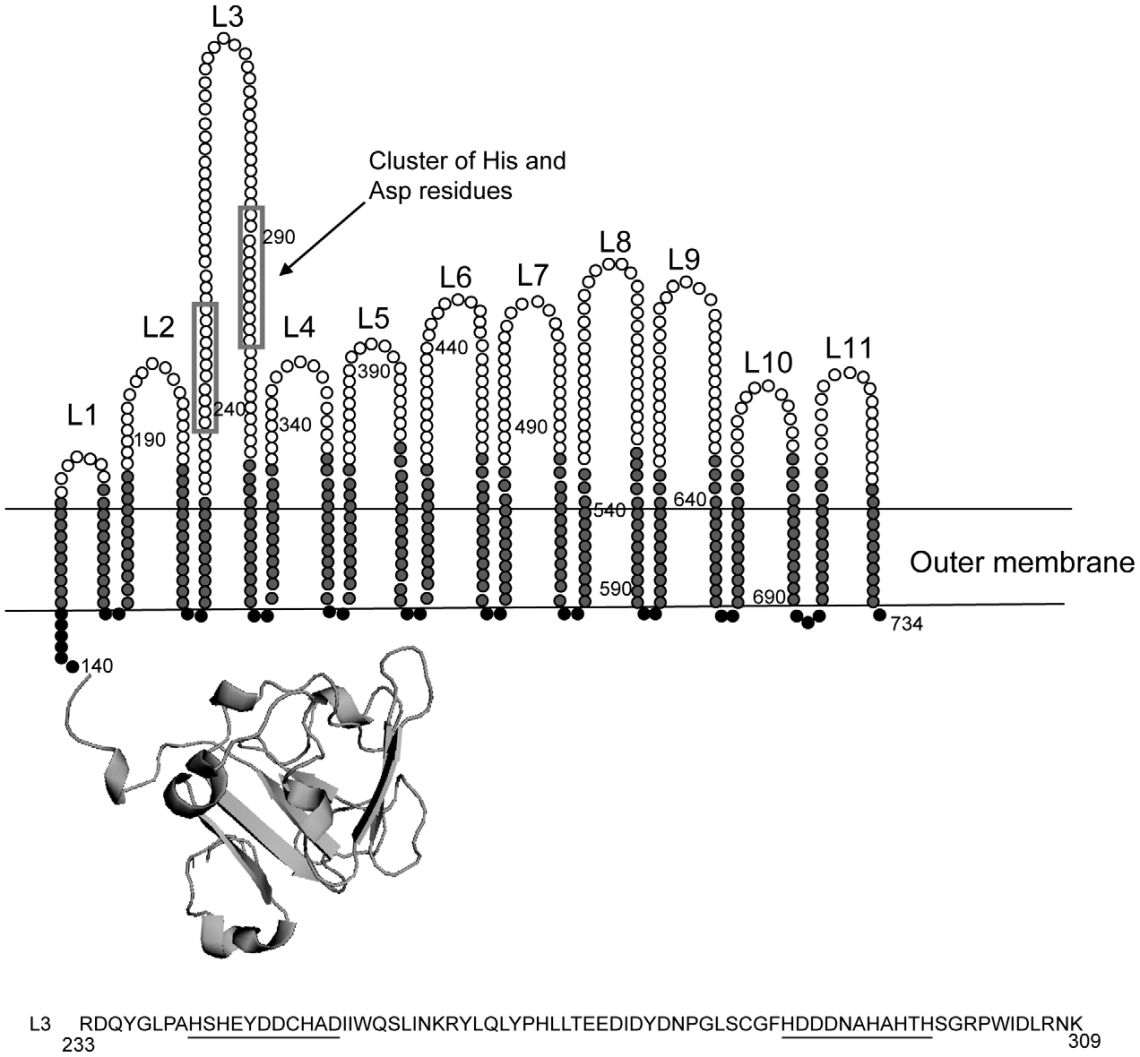
Topology model of ZnuD. The 22 β-strands are colored light grey, the 11 extracellular loops are white and the periplasmic turns are black. The histidine/aspartic acid stretches are boxed. The plug domain was modeled based on known Tdf structures. The amino acid sequence of loop 3 is shown with the cysteines in bold and the His/Asp-rich stretches underlined. Numbers indicate amino acid positions in the sequence of mature ZnuD.

**Figure 3:**
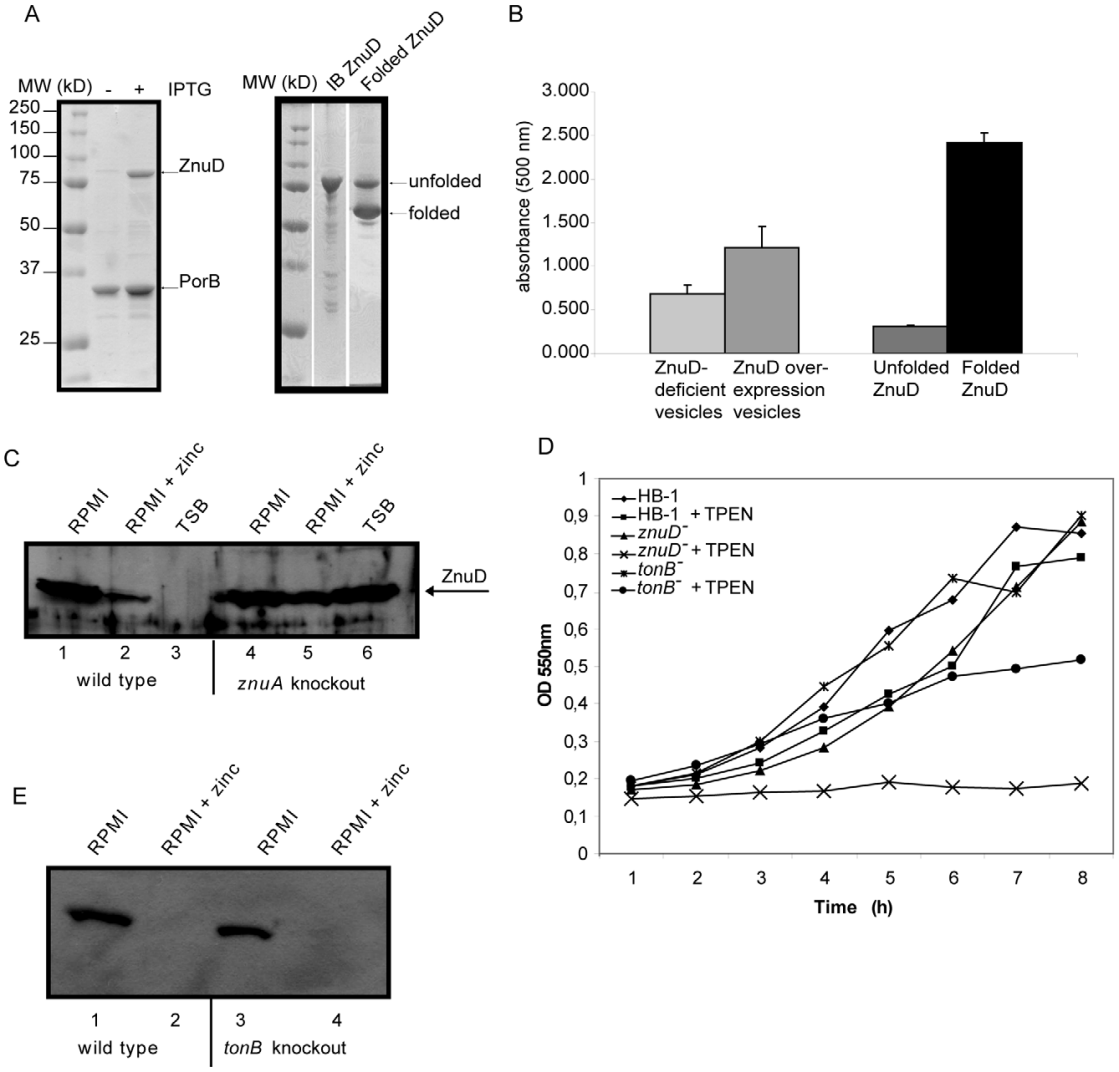
Zinc binding and transport by ZnuD. (A) Coomassie-stained SDS-PAGE gels showing the protein content of the OMVs (left) and purified ZnuD preparation (right) used in the PAR-binding assay. OMVs were isolated from strain CE1523 carrying pEN11-*znuD* that was either induced or not with IPTG for *znuD* expression as indicated. Purified ZnuD from inclusion bodies (IB ZnuD) was refolded in vitro (Folded ZnuD). The folded ZnuD sample was not heated before SDS-PAGE and the positions of folded and unfolded ZnuD are indicated. (B) Binding of zinc to OMVs either or not containing ZnuD and to purified ZnuD that was either folded or not was measured in a PAR competition assay. Shown are the normalized values of the absorption at 500 nm of five independent measurements. (C) Western blot of whole cell lysates of strain HB-1 (lanes 1–3) and the *znuA* mutant (lanes 4–6) grown in RPMI (lanes 1 and 4), RPMI with 500 nM ZnSO_4_ (lanes 2 and 5) or TSB (lanes 3 and 6). (D) Representative growth curves (n = 5) of the *znuD* and *tonB* knockout strains and their parent strain in response to zinc limitation. Cultures contained either 0 or 0.3 µM TPEN. (E) Western blot of whole cell lysates of strain HB-1 (lanes 1–2) and the *tonB* mutant (lanes 3–4) grown in RPMI (lanes 1 and 3) or RPMI with 500 nM ZnSO_4_ (lanes 2 and 4).

Since the expression of *znuD* is regulated by the availability of zinc, it seemed likely that ZnuD acts as a receptor for zinc or a zinc-containing compound. We first analyzed the amino acid sequence and constructed a topology model of the barrel domain of ZnuD using the PROFtmb program at www.rostlab.org [21] and a 3D structural model of the plug domain based on known structures of TonB-dependent receptors using 3D-jigsaw [22] (Figure 2). ZnuD contains two cysteine residues in the putative extracellular loop L3. When these cysteines form a disulfide bond, they bring two stretches of amino acid residues, both rich in histidine and aspartic acid residues, in close proximity (Figure 2). The resulting His- and Asp-rich domain could be of functional importance, since also in the periplasmic zinc-binding protein ZnuA of *E. coli*, a stretch of His and Asp residues is involved in binding its ligand [23]. Thus, we considered the possibility that ZnuD binds free zinc and transports it into the periplasm. To test this hypothesis, we first determined whether ZnuD could bind zinc. To this end, *N. meningitidis* strain CE1523, an H44/76 derivative that lacks porin PorA and the polysaccharide capsule, was transformed with a plasmid carrying *znuD* under the control of an isopropyl-β-D-1-thiogalactopyranoside (IPTG)-inducible promoter. The resultant strain was grown with and without IPTG, and outer membrane vesicles (OMVs) were isolated (Figure 3A, left panel) and compared for their capacity to compete with 4-(2-pyridylazo)resorcinol (PAR) for binding zinc. In the presence of OMVs containing ZnuD, ∼40% more free PAR was measured than in the presence of OMVs lacking ZnuD, indicating that ZnuD is capable of binding zinc (Figure 3B). To demonstrate the involvement of ZnuD in binding zinc directly, ZnuD was produced in *E. coli* in inclusion bodies, which were isolated, and the protein was folded in vitro into its native conformation. Like many other outer membrane proteins [24], ZnuD displays heat modifiability, *i.e.* the denatured form has a lower electrophoretic mobility than the correctly folded form, a property that was used to monitor proper folding (Figure 3A, right panel). The native protein was then tested alongside the unfolded protein in the PAR competition assay. The folded protein indeed competed with PAR for zinc while the unfolded protein did not (Figure 3B) showing the specificity of the reaction.

If ZnuD is indeed involved in the uptake of free zinc, one would expect a higher external zinc concentration to be required to repress expression of *znu* genes in the *znuD* mutant than in the wild-type strain. To test this idea, the *znuD* mutant and its parent strain were grown in RPMI medium supplemented with 0.5 µM ZnSO_4_, which largely, but not completely represses *znuD* expression in the wild-type strain (Figure 1D). The relative levels of *znuD* and *znuA* mRNA were then measured by RT-qPCR. Of note, the *znuD* mutant still contains the first 437 nucleotides of the *znuD* gene and 100 nucleotides thereof were used for the detection of *znuD* gene expression. In the *znuD* mutant, there was 18.6±1.1-fold more *znuD* and 7.4±1.1-fold more *znuA* expressed compared to the parent strain, showing that indeed the intracellular zinc concentration in the *znuD* mutant is lower than that in the parent strain under the applied growth conditions. Also, Western-blot analysis showed that a *znuA* knockout strain produced high levels of ZnuD in the presence of zinc, confirming that ZnuA is required to sustain sufficient zinc levels in the cell (Figure 3C).

Finally, we tested whether expression of *znuD* offers any growth advantage under zinc limitation. To deplete the internal zinc stores, the bacteria were first grown overnight on RPMI plates, supplemented with 100 µM ferric chloride to prevent iron depletion. Subsequently, they were inoculated in RPMI supplemented or not with TPEN. In the absence of TPEN, growth of the bacteria was only marginally affected by the absence of ZnuD, but, in contrast to the parental strain, the *znuD* mutant failed to grow when 0.3 µM TPEN was added to the medium (Figure 3D). These results indicate that *znuD* expression is indeed beneficial to the cells when the available zinc is limiting.

We also assessed whether TonB is required for ZnuD-mediated zinc acquisition. In growth experiments, a *tonB* knockout strain grew less well than the parent but better than the *znuD* mutant in the presence of TPEN (Figure 3D). However, in RT-qPCR experiments, we did not observe increased expression of *znuA* or *znuD* after growth of the *tonB* mutant in RPMI supplemented with 0.5 µM ZnSO_4_ and, consistently, we could not detect more ZnuD under these conditions by Western-blot analysis (Figure 3E). Together, these results suggest that TonB facilitates the uptake of free zinc through ZnuD, but that uptake can take place also independently of TonB.

### Vaccine potential of ZnuD

**Figure 4:**
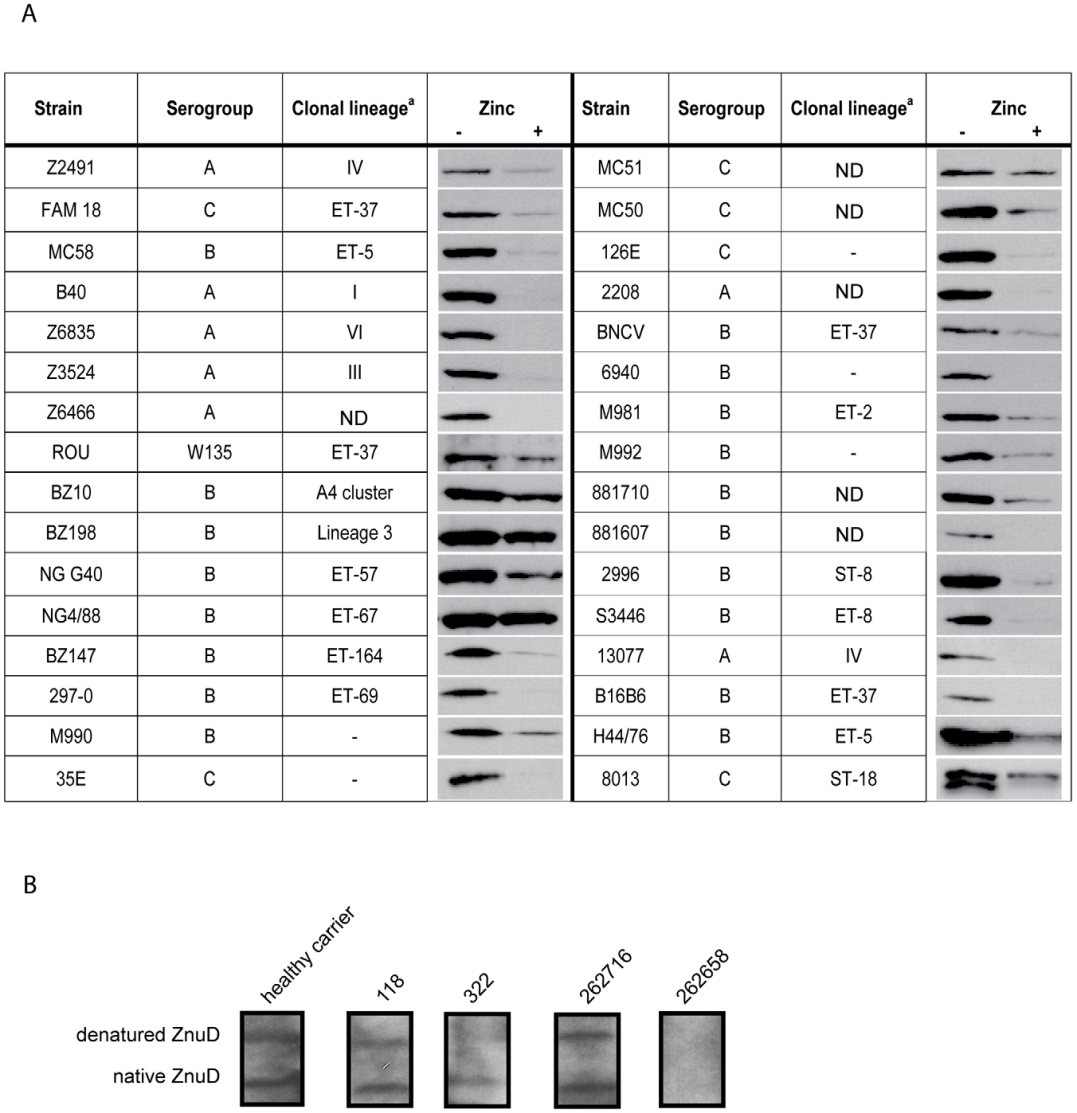
ZnuD synthesis in meningococcal isolates and in vivo. (A) Western blots of cell lysates of the indicated strains grown in RPMI with or without added zinc. ^a^ Clonal group designations taken from [40]; –, the strain was typed by Multi-Locus Enzyme Electrophoresis but could not be assigned to a specific clone; ND, not determined. (B) Reaction of human sera with ZnuD on Western blots. The blots contained both native and denatured ZnuD. The specific patient sera used are indicated above the lanes and have been described previously [10], [25]. Also an example of a non-reactive patient serum is shown at the right.

To investigate whether ZnuD could be a candidate component for a universal *N. meningitidis* vaccine, we first studied its distribution among various *N. meningitidis* isolates. ZnuD homologs were found in all the available *N. meningitidis* genome sequences and they showed a strikingly high 97–99% amino acid identity in the mature part of the protein (Figure S1, Supporting Information). The sequence differences are scattered throughout the protein and are not clustered in predicted extracellular loop regions, which are often antigenically variable in *Neisseria* outer membrane proteins. We subsequently analyzed the presence of ZnuD in a panel of 32 different *N. meningitidis* isolates from different serogroups and different clonal lineages. The strains were grown in RPMI medium supplemented or not with 0.5 µM ZnSO_4_, and whole cell lysates were analyzed by Western blotting with the antiserum raised against ZnuD of strain H44/76. ZnuD was detected in all strains tested and in all cases *znuD* expression was repressed in the presence of zinc, albeit to different extents (Figure 4A).

To investigate whether ZnuD is expressed in the human host, we tested sera from convalescent patients, healthy carriers and non-carriers [10], [25] for the presence of antibodies that recognize ZnuD on Western blots. The reactivity of the sera was tested against both the denatured and the refolded form of the ZnuD protein (Figure 3A, right panel). We could detect ZnuD-specific antibodies in sera from most convalescent patients and healthy carriers tested (examples are shown in Figure 4B), although also some sera were negative (e.g. serum 262658 in Figure 4B). The positive sera reacted with both the denatured protein and the protein folded in vitro into its native conformation although the relative intensity of the reaction with the two forms of the protein varied (Figure 4B). No ZnuD-specific antibodies were detected in the sera from non-carriers (results not shown).

Next, we immunized 10 mice with OMVs from a strain overexpressing this protein (Figure 3A) and tested the resultant pooled sera for the presence of bactericidal antibodies. Routinely, we perform serum bactericidal assays on bacteria grown in TSB medium; however, under these conditions *znuD* is not expressed. Therefore, we tested the sera for bactericidal activity on a derivative of strain H44/76 that expressed *znuD* from an IPTG-inducible promoter and compared cultures grown with and without 1 mM IPTG. The bactericidal titers of the pooled sera were <1∶100 against bacteria not producing ZnuD, but 1∶1042 against bacteria producing ZnuD. Titers in pre-immune sera were <1∶100 independent of whether ZnuD was produced or not. These data clearly show that ZnuD is able to elicit bactericidal antibodies. Thus, since ZnuD is highly conserved among *N. meningitidis* isolates and elicits bactericidal antibodies, it might be an attractive vaccine component.

### ZnuD homologs in other bacteria

Genes encoding the high-affinity ZnuABC uptake system for zinc have been identified in many bacteria, but the involvement of an outer membrane receptor in zinc acquisition has not been described so far. To investigate whether an outer membrane receptor might be more generally associated with zinc acquisition, we searched for ZnuD homologs in other pathogenic bacteria by performing BLAST searches at NCBI. ZnuD homologs with high sequence similarity (∼96% identity) were found in *Neisseria gonorrhoeae* strain FA1090 (locus NGO_1205) and other *N. gonorrhoeae* strains. Homologs of ZnuD were also found in other pathogenic bacteria, including *Moraxella catarrhalis*, *Haemophilus parasuis*, *Mannheimia haemolytica*, *Acinetobacter baumannii*, *Pasteurella multocida*, *Bordetella pertussis*, and *Actinobacillus pleuropneumoniae* (Table 1). All these ZnuD homologs contain the His- and Asp-rich regions suspected to be involved in zinc binding (Figure S2, Supporting Information). Interestingly, in *B. pertussis* the *znuD* homolog is located adjacent to a gene cluster containing homologs of the *znuABC* and *zur* genes, again indicating a functional relationship between these genes. Thus, it appears that outer membrane receptor-mediated zinc acquisition is not specific for *N. meningitidis*, but is more common among pathogenic Gram-negative bacteria.

**Table 1:** Identity and similarity of ZnuD with its homologs.

Strain	Accession number	% identity	% similarity
*Moraxella catarrhalis*	AAU94646	41	58
*Haemophilus parasuis*	YP_002474986	40	58
*Mannheimia haemolytica*	AAK29743	46	63
*Acinetobacter baumannii*	YP_001651932	41	61
*Pasteurella multocida*	NP_246018	44	59
*Bordetella pertussis*	NP_881648	35	51
*Actinobacillus pleuropneumoniae*	YP_001651932	41	57
*Neisseria gonorrhoeae*	YP_208276	96	97

## Discussion

The Gram-negative bacterial outer membrane forms a protective barrier that protects the bacteria against harmful compounds from the environment, including antibiotics, detergents and digestive enzymes. By the presence of porins, it functions as a molecular sieve that allows for the passage of hydrophilic solutes with molecular weights up to ∼600 Da by passive diffusion. Most nutrients can pass the outer membrane via the porins. However, passive diffusion is effective only when the external solute concentration is high. The bacteria may also require nutrients that are present in the environment in low concentrations or that are too large to pass through the porins. For such cases, the bacteria have developed active transport systems in the outer membrane that depend on a Tdf receptor with high affinity for its ligand and on the TonB complex that delivers the electrochemical energy of the proton gradient across the inner membrane to the transport process across the outer membrane [3]–[5].

Most Tdf members studied to date are involved in iron acquisition [1], [2], a notable exception being BtuB protein of *E. coli*, which mediates the uptake of vitamin B12 [26]. Since the free iron concentration in the human host is too low to support bacterial growth, efficient iron-acquisition systems constitute important virulence factors of pathogenic bacteria. These bacteria usually can use multiple iron sources including siderophores produced by themselves or by other micro-organisms, heme, and iron-transport and -storage proteins of the host. For each iron source, the bacteria require a specific receptor. However, based on the available genome sequences, the number of Tdf receptors in a bacterium can be very high. For example, *Pseudomonas aeruginosa* may contain up to 37 different Tdf receptors [27] suggesting that at least some of these receptors might have functions other than iron or vitamin B12 transport. Indeed, in *Caulobacter crescentus*, Tdf receptors have been described that are involved in the uptake of carbon sources [28], [29]. *C. crescentus* thrives in nutrient-poor fresh-water lakes where receptor-mediated active transport across the outer membrane offers a solution to acquire sufficient nutrients. Also, very recently, a receptor for nickel has been described in *Helicobacter mustelae* [30].

Here, we describe for the first time a Tdf receptor involved in the acquisition of zinc. Like the levels of free iron in the human host, those of free zinc are most likely too low to sustain bacterial growth. For example, although the total concentration of zinc in human serum is approximately 13 µM, the vast majority of it is bound by serum proteins such as albumin [31]. In addition, the human host responds to infections by the production of metallothioneins and calprotectin, which reduce the availability of zinc and other metals to the invading pathogens [6], [7]. The high-affinity ZnuABC system for the uptake of zinc across the inner membrane has been identified in many bacteria, including *Neisseria gonorrhoeae* [32] and *Salmonella enterica* [33] where it was shown to be associated with virulence [33], [34]. Likewise, the outer membrane receptor for zinc acquisition identified here, ZnuD, is presumably important for virulence, which, however, is difficult to establish in *N. meningitidis*, for which a suitable animal model is lacking.

Our results show that ZnuD can bind zinc and that *znuD* expression facilitates the uptake of free zinc at low zinc concentrations. Although ZnuD is a Tdf member, TonB appeared not to be required for ZnuD-mediated zinc uptake. However, it is entirely possible that ZnuD, besides its function as a receptor for free zinc, could additionally recognize a complexed form of zinc, which may be available in the respiratory tract, in serum and/or in cerebral fluid. If that is the case, we expect that the TonB system will be needed for the acquisition of zinc from such ligand.

Previously, *znuD* expression was reported to be induced in the presence of active complement [35]. In that microarray study, the expression profiles were compared of *N. meningitidis* strain Z5463 grown in the presence of serum that was either inactivated or not by heat. Expression of *znuD* (locus tag NMA1161) was found 23-fold de-repressed in the presence of the untreated serum. A possible explanation for this observation is the presence in serum of albumin, which is known to chelate zinc [31]. Heat treatment of serum will probably release zinc from albumin, which could repress *znuD* expression, while *znuD* would be expressed in bacteria exposed to untreated serum where zinc is chelated. Consistent with this explanation is the observation in the same microarray study that also the genes with locus tags NMA1137 and NMA1138, which encode paralogs of the ribosomal proteins L36 and L31 that are induced in many bacterial species under zinc limitation [36], were strongly induced in the presence of the untreated serum [35].

*N. meningitidis* normally lives as a commensal on the mucosal surfaces in the upper respiratory tract. Zinc is found on mucosal surfaces, but the total zinc concentrations or the amount of free zinc present are not known. However, it is intriguing that ZnuD homologs were particularly found in bacterial species residing in the respiratory tract of humans and animals. Probably, the unbound zinc concentration in the mucosal layers of the respiratory tract is too low to allow sufficient passive diffusion through the porins and, therefore, ZnuD may become essential for bacterial growth and survival particularly in this niche. This hypothesis is strengthened by the detection of specific antibodies against ZnuD in the serum from healthy carriers, which indicates that ZnuD is expressed where the bacteria normally reside, the nasopharynx.

As a pathogen, *N. meningitidis* can enter the bloodstream and cause sepsis and meningitis with a high mortality rate. A vaccine against serogroup B meningococci is not available, because the corresponding capsular polysaccharide is poorly immunogenic. Outer membrane proteins are being studied as alternative vaccine components, but these studies are frustrated by the high antigenic variability of the major outer membrane proteins. Since ZnuD is highly conserved among *N. meningitidis* isolates and elicits bactericidal antibodies, it might be an attractive vaccine component, particularly in combination with other minor outer membrane proteins due to the synergistic bactericidal activity of antibodies against such antigens [37].

## Materials and Methods

### Bacterial strains and growth conditions

Except when indicated otherwise, experiments were performed with the unencapsulated *N. meningitidis* strain HB-1 [38] and mutants thereof. Strain CE1532 was constructed from strain CE2001, a *porA*-deficient derivative of H44/76 [39], by inactivating the capsule locus similarly as described for HB-1 [38]. *N. meningitidis* was grown on GC agar (Oxoid) plates containing Vitox (Oxoid) and antibiotics when appropriate (kanamycin, 100 µg/ml; chloramphenicol, 10 µg/ml) in candle jars at 37°C. Liquid cultures were grown in TSB (Difco) or in RPMI (Sigma) at 37°C with shaking. *E. coli* strains DH5α and TOP10F′ (Invitrogen) were used for routine cloning. *E. coli* was propagated on Luria-Bertani (LB) medium supplemented when appropriate with 100 µg/ml ampicillin, 50 µg/ml kanamycin, or 25 µg/ml chloramphenicol.

### Construction of plasmids and mutants

Primers used are listed in Table S1 (Supporting Information). The *znuD* gene without the signal sequence-encoding part was amplified from chromosomal DNA of strain H44/76 by PCR using the primers 0964-F and 0964-R and cloned into pCRII-TOPO (Invitrogen), generating pCRII-*znuD*. From there, it was subcloned into pET11a (Novagen) using NdeI/BamHI restriction, resulting in plasmid pET11a-*znuD*.

To obtain a *znuD* deletion construct, a kanamycin-resistance gene cassette was amplified by PCR with the primers Kan-R and Kan-F from pCR2.1-Kan/Dus [40] and cloned after MluI and BsrGI digestion into pCRII-*znuD* digested with the same enzymes. In the resulting construct, pCRII-*znuD*::kan, the kanamycin-resistance cassette substitutes for the region between base pairs 437 and 1344 of *znuD*. The *znuD*::kan construct was amplified with primers 0964-R and 0964-F and used to transform strain HB-1 to generate a *znuD* mutant.

For regulated expression of *znuD*, the entire *znuD* gene from H44/76 was amplified with primers ZnuD-F and ZnuD-R. The PCR product was cloned via pCRII-TOPO into the neisserial replicative plasmid pEN11-pldA [40] using NdeI and AatII restriction. In the resulting plasmid, pEN11-*znuD*, the *znuD* gene is under control of an IPTG-inducible tandem *lac/tac* promoter.

To obtain a *tonB* knockout construct, DNA fragments upstream and downstream of *tonB* (NMB1730) were amplified using primer couples tonB-1/tonB-2 and tonB-3/tonB-4. The two fragments were each cloned into pCRII-TOPO and then ligated together using the AccI restriction site introduced via the primers and the SpeI site present in the vector. The AccI site was used to insert the chloramphenicol transacetylase gene obtained from pKD3 [41] with primers P1 and P2. The resulting construct was amplified using primers tonB-1 and tonB-4 and the PCR product was used to transform *N. meningitidis* HB-1 to generate a *tonB* mutant. The *zur* and *znuA* genes were knocked out following the same strategy. The primer couples used to obtain the upstream and downstream fragments were zur-1/zur-2 and zur-3/zur-4, and znuA-1/znuA-2 and znuA-3/znuA-4.

### SDS-PAGE and Western blot analysis

Whole cell lysates were prepared from liquid cultures by resuspending cell pellets in sample buffer. Proteins were separated by routine SDS-PAGE or by semi-native SDS-PAGE, which allows for analysis of outer membrane proteins in their native β-barrel conformation [40]. After electrophoresis, the gels were either stained with Coomassie brilliant blue or the proteins were transferred to nitrocellulose membranes (Protran) using a wet transfer system (Biorad) in 25 mM Tris-HCl, 192 mM glycine, 20% methanol. Membranes were blocked for 1 h in PBS containing 0.1% Tween 20 and 0.5% Protifar (Nutricia). Blots were incubated with antibodies in blocking buffer. Antibody binding was detected by using peroxidase-conjugated goat anti-rabbit or anti-human IgG secondary antibodies (Biosource) and enhanced chemiluminescence detection (Pierce).

### Isolation and refolding of recombinant ZnuD

*E. coli* BL21(DE3) (Invitrogen) containing pET11a-*znuD* was grown in LB to an optical density at 600 nm of 0.6 after which 1 mM IPTG was added and growth was continued for 2 h. ZnuD accumulated in inclusion bodies, which were isolated as described [24]. The inclusion bodies were dissolved in 20 mM Tris-HCl, 100 mM glycine, 6 M urea (pH 8.3), and residual membranes were removed by centrifugation for 1 h at 200,000 *g*. The protein was then refolded into its native conformation by diluting this stock solution 20-fold in refolding buffer containing 55 mM Tris-HCl, 0.21 mM sodium chloride, 0.88 mM potassium chloride, 880 mM L-arginine and 0.5% 3-dimethyldodecylammoniopropane-sulfonate (SB-12) (Fluka), pH 7.0. After refolding overnight, the sample was dialyzed to 55 mM Tris-HCl, 0.21 mM sodium chloride, 10 mM L-arginine, and 0.5% SB-12, pH 6.5. The protein solution was filtered and stored at 4°C. Proper folding was monitored by semi-native SDS-PAGE where the folded protein has a higher electrophoretic mobility than the denatured protein.

### Immunizations

Preparative SDS-PAGE was used to purify ZnuD from inclusion body preparations. After staining the gel with Coomassie brilliant blue, the band corresponding to ZnuD was electro-eluted (Biorad) and used to immunize rabbits at Eurogentec.

To generate OMVs, strain CE1523 containing pEN11-*znuD* was grown in the presence or absence of 1 mM IPTG. OMVs were prepared by deoxycholate extraction and used to immunize mice as described [10]. Sera from ten mice per group were collected after 42 days and pooled.

### Ethics statement

All animal experimentations were performed in compliance with the current Belgian legislation related to the protection and well-being of animals and the European directive 86/609/CEE related to the protection of vertebrate animals used for experimental or other scientific purposes. The experimental protocols have been approved by GSK Biologicals Ethical Commission for Animal Experimentation. All experimental procedures were conducted at the GSK Belgium facilities, which has a full AAALAC (Association for Assessment and Accreditation of Laboratory Animal Care) accreditation since October 25th 2004.

### RT-qPCR

RT-qPCR was performed using a 7900HT Fast Real-Time PCR System and SYBR green master mix (Applied Biosystems). Total RNA was isolated using Trizol (Invitrogen) and further purified with nucleospin RNA II columns (Macherey-Nagel) and treated with Turbo DNA-Free (Ambion) to yield DNA-free RNA. cDNA was generated from 1 µg RNA using transcriptor High fidelity cDNA synthesis kit (Roche). As a control, samples without the reverse transcriptase were tested in parallel. PCRs were performed in triplicate. The primers used are listed in Table S1. A melting plot was performed to ensure that the signal originated from the specific amplicon. Data analysis was performed using the comparative cycle threshold method (Applied Biosystems) to determine relative expression levels. The *rmpM* transcript was used to normalize all data.

### ICP-MS

Total zinc concentrations in RPMI medium were measured by ICP-MS using an X Series 2 ICPMS (Thermo Scientific). Filtered (0.22-µm) medium was acidified with HNO_3_ (suprapure, Merck) prior to the measurements.

### Zinc binding assay

In the PAR (Fluka) competition assay, the orange color of a PAR/zinc complex changes towards yellow in the presence of a protein that releases zinc from PAR. The assay was performed as described [42] using 30 µM ZnSO_4_ and 20 µg OMVs. The assay with the refolded protein as competitor was carried out in 55 mM Tris-HCl, 0.21 mM sodium chloride, 10 mM L-arginine, and 0.5% SB-12, pH 6.5; in this assay, 50 µM PAR, 20 µM ZnSO_4_, and 16 µg ZnuD were used.

### Serum bactericidal assay

Wild type H44/76 carrying pEN11-*znuD* was inoculated from overnight grown plates in TSB with 125 µM FeCl_3_ with or without 1 mM IPTG in shaking flasks for 3 h at 37°C until an optical density at 550 nm of 0.5 was reached. Serum bactericidal assays were performed as described [10]. Bactericidal titers are defined as the highest serum dilution yielding >50% killing.

### Accession numbers

Accession numbers for the meningococcal proteins described in this study in Genbank are: ZnuD (NMB0964), AAF62323; ZnuA (NMB0586), AAF41014; ZnuB (NMB0587), AAF41015; ZnuC (NMB0588), AAF41016; Zur (NMB1266), AAF41643; TonB (NMB1730), AAF42075. Other accession numbers are provided in Table 1.

## Supporting Information

Figure S1 Alignment of meningococcal ZnuD homologs. Aligned is the amino acid sequence of *N. meningitidis* strain MC58 with those of strains 053422, FAM18 and Z2491, and the carrier strains α14 and α153. The signal sequence, TonB box (Tb), plug domain, surface-exposed loops and the transmembrane domains (Tm) are marked above the sequence and the His- and Asp-rich stretches are underlined.
(0.07 MB PDF)

Figure S2 Alignment of ZnuD homologs. Aligned are the amino acid sequences of various pathogens. The histidine- and aspartic acid-rich stretches are highlighted in grey.
(0.05 MB PDF)

Table S1 Primers used in this study.
(0.05 MB DOC)
