# Supplementary material for: An Outer Membrane Receptor of Neisseria meningitidis Involved in Zinc Acquisition with Vaccine Potential
Source: PLoS Pathog. 2010 Jul 1;6(7):e1000969. doi: 10.1371/journal.ppat.1000969 (PMC2895646; doi:10.1371/journal.ppat.1000969)
Supplement: Figure S1 — Alignment of meningococcal ZnuD homologs. Aligned is the amino acid sequence of N. meningitidis strain MC58 with those of strains 053422, FAM18 and Z2491, and the carrier strains α14 and α153. The signal sequence, TonB box (Tb), plug domain, surface-exposed loops and the transmembrane domains (Tm) are marked above the sequence and the His- and Asp-rich stretches are underlined. (0.07 MB PDF) [file ppat.1000969.s001.pdf]

Figure S1

```
              <    Signal sequence   >          <Tb > <    plug domain
MC58        1 MAQTTLKPIVLSILLINTPLLAQAHETEQSVDLETVSVVGKSRPRATSGLLHTSTASDKI 60
O53322        ...............................G....T.......................
Z2491         .....................S...G.....G............................
FAM18         ............................................................
α14           ............................................................
α153          ...........................DR...............................

                       plug domain
MC58       61 ISGDTLRQKAVNLGDALDGVPGIHASQYGGGASAPVIRGQTGRRIKVLNHHGETGDMADF 120
O53422        ............................................................
Z2491         ............................................................
FAM18         L...........................................................
α14           ............................................................
α153          ............................................................

                    plug domain                  >< Tm1    >
MC58      121 SPDHAIMVDTALSQQVEILRGPVTLLYSSGNVAGLVDVADGKIPEKMPENGVSGELGLRL 180
O53422        ............................................................
Z2491         .........S..................................................
FAM18         ............................................................
α14           ............................................................
α153          ............................................................

              <loop1>< Tm2     >  <     Tm3  ><       loop2     ><    Tm4
MC58      181 SSGNLEKLTSGGINIGLGKNFVLHTEGLYRKSGDYAVPRYRNLKRLPDSHADSQTGSIGL 240
O53422        .....................................................K......
Z2491         ............................................................
FAM18         ............................................................
α14           ............................................................
α153          ............................................................

                 >  <  Tm5   ><                loop3
MC58      241 SWVGEKGFIGVAYSDRRDQYGLPAHSHEYDDCHADIIWQKSLINKRYLQLYPHLLTEEDI 300
O53422        ..........A................................................V
Z2491         ..........A.................................................
FAM18         ............................................................
α14           ...........................................................V
α153          ..........A.................................................

                                              ><     Tm6   >  <  Tm7     >
MC58      301 DYDNPGLSCGFHDDDNAHAHTHSGRPWIDLRNKRYELRAEWKQPFPGFEALRVHLNRNDY 360
O53422        ......................N.K...................L...............
Z2491         ...............D....A.N.K...................................
FAM18         ...............D............................................
α14           ...............D....A.N.K...................................
α153          ...............D....A.N.K...................................

              <    loop4      ><   Tm8     >  <   Tm9     ><    loop5
MC58      361 RHDEKAGDAVENFFNNQTQNARIELRHQPIGRLKGSWGVQYLQQKSSALSAISEAVKQPM 420
O53422        H...............K...........................................
Z2491         ..........................................G........T........
FAM18         ............................................................
α14           ...................................................T........
α153          ...................................................T........
```

```
                    ><   Tm10     >  <   Tm11   ><        loop6
MC58       421 LLDNKVQHYSFFGVEQANWDNFTLEGGVRVEKQKASIQYDKALIDRENYYNHPLPDLGAH 480
O53422         .................E................................KQ........
Z2491          .....................................R......................
FAM18          .....................................R......................
α14            .....................................R......................
α153           .....................................R......................

                  ><   Tm12    >  <    Tm13  ><       loop7               >
MC58       481 RQTARSFALSGNWYFTPQHKLSLTASHQERLPSTQELYAHGKHVATNTFEVGNKHLNKER 540
O53422         ............................................................
Z2491          ............................................................
FAM18          ............................................................
α14            ............................................................
α153           ............................................................

               <   Tm14    >  <  Tm15    ><               loop 8
MC58       541 SNNIELALGYEGDRWQYNLALYRNRFGNYIYAQTLNDGRGPKSIEDDSEMKLVRYNQSGA 600
O53422         ............................................................
Z2491          ............................................................
FAM18          ............................................................
α14            ............................................................
α153           ............................................................

               ><    Tm16    >  <    Tm17  ><          loop9
MC58       601 DFYGAEGEIYFKPTPRYRIGVSGDYVRGRLKNLPSLPGREDAYGNRPFIAQDDQNAPRVP 660
O53422         ............................................................
Z2491          ...............................................L...A........
FAM18          ............................................................
α14            ............................................................
α153           ............................................................

               ><    Tm18   >  <   Tm19   ><   loop10  ><   Tm20    >  <
MC58       661 AARLGFHLKASLTDRIDANLDYYRVFAQNKLARYETRTPGHHMLNLGANYRRNTRYGEWN 720
O53422         .....V......................................................
Z2491          .....V......................................................
FAM18          .....V......................................................
α14            .....V......................................................
α153           .....V......................................................

               Tm21   ><      loop 11   ><  Tm22   >
MC58       721 WYVKADNLLNQSVYAHSSFLSDTPQMGRSFTGGVNVKF 758
O53422         ......................................
Z2491          ......................................
FAM18          ......................................
α14            ......................................
α153           ......................................
```

Figure S1. Alignment of meningococcal ZnuD homologs. Aligned is the amino acid sequence of *N. meningitidis* strain MC58 with those of strains 053422, FAM18 and Z2491, and the carrier strains α14 and α153. The signal sequence, TonB box (Tb), plug domain, surface-exposed loops and the transmembrane domains (Tm) are marked above the sequence and the His- and Asp-rich stretches are underlined.
