# Supplementary material for: An Outer Membrane Receptor of Neisseria meningitidis Involved in Zinc Acquisition with Vaccine Potential
Source: PLoS Pathog. 2010 Jul 1;6(7):e1000969. doi: 10.1371/journal.ppat.1000969 (PMC2895646; doi:10.1371/journal.ppat.1000969)
Supplement: Figure S2 — Alignment of ZnuD homologs. Aligned are the amino acid sequences of various pathogens. The histidine- and aspartic acid-rich stretches are highlighted in grey. (0.05 MB PDF) [file ppat.1000969.s002.pdf]

Figure S2

```
N. meningitidis     MAQTTL----KPIVLS-ILLI-N-TPLLAQAHE--TEQSVDLETVSVVGKSRPRATSGLLHTSTAS
M. haemolytica      MLKK------NYLTVS-ILLAIS-GVGYA--------NEISLETITVDGNTPSTKGKLLGGELNSN
A. baumanii         MLNKSK------LFLALITLGAS-KIL-L-AA---EGPVTTLNTIVLTAQSDELGSELLGKSLNVS
M. catarrhalis      MKVTMI---KKPLAC-AILATFS-MPMLAEANLKD-KPTVILDGVSITSLADQNTEFGVNHSKTVS
H. parasuis         MINNRTTEQQNNRTT-AFSLAFS-LLLCCLGI---NAEQLELDEISVMGKVPE---GNSISFLKVS
P. mutocida         MRKI------SYLSLCVISALYS-QLAVAQSPLKNTSEHIELEPIFVNTLIESREGAPLGGRLMAS
A. pleuropneumoniae MFNKKL------LAV-LISAQFS-PLV-W-AN---NNDVAVLDEVSVVGSTPSISQGSEVTLLKVS
B. pertussis        MRFER-----HPLSAA-LALALAWQGAHAQASADGTPEAATLAPITVSAS-PL-A--GDLDSMTAP
                    *                :                       *  : :                  :

N. meningitidis     DKIISGDT-LRQKAVNLGDALDGVPGIHASQYGGGASAPVIRGQTGRRIKVLNHHGETGDMADFSP
M. haemolytica      ESVVDEKN-LKQGSITLGNALSGELGIHSSQFGGGASTPIIRGQESKRAKILQNNGENLDMSGMSP
A. baumanii         NQFIDTSK-LKQRSTTLGDALGTELGIHSNQYGGGASAPIIRGQEGKRIKVLQNNADVLDMSNMSP
M. catarrhalis      GITVSKEQ-LQQRATTLGDALAGELGVHSNHFGGGASAPIIRGQEGKRLKILQNGSEVVDMSGLSP
H. parasuis         DAIIDGEK-FKNRSATLGNALSSELGVHSTPFGGGASAPIIRGQEGVRVKILQNNADVVDMSNISP
P. mutocida         EKIIPAYS-LKQRGSNLGDALSSELGIHASQFGGGASAPVIRGQEGKRIKVLSSGNETLDMSAMSP
A. pleuropneumoniae DKIIAGKE-FKKRSATLGNALAAELGVHSNPFGGGASKPIIRGQEGARIRILQNGSDVIDMSNLSP
B. pertussis        AAVLEGDQLLLRRQGTLGDTLDGLPGVHADTFGGGASRPVIRGQTAPRVKVLSDGSELMDASAISP
                         : .   .**::*    *:*:  :***** *:**** . * ::*.       :  * : :**

N. meningitidis     DHAIMVDTALSQQVEILRGPVTLLYSSGNVAGLVDVADGKIPEKMPENGVSGELGLRLSSGNLEKL
M. haemolytica      DHAVTVDALLAKRIEILRGPTTLLYSAGNTAGVINVVDNKIPTAIPEKGYEGQFGVRFGSASKERL
A. baumanii         DHAVTVEPSLAKSIEIIRGASTLLYSSNSAAGVVNVIDYKIPTQMPQDGLEGNTTLRFNTGSNEKL
M. catarrhalis      DHAIAVDTTLAKQVEIVRGSGALLYASGNSAGVVNVVDDKIPSKLPSK-LQGDVTVRLSSANREKL
H. parasuis         DHAITADTLLANQVEILRGASTLLYASSSPAGIVNIVDQRIPNKMPKKGYEVTLSSRFDTASKEKV
P. mutocida         DHAVAVDSLLAKKVEILRGANTLLYSSGNAAGVVNVVDNKIPTAE-VVGVEGEVGLRTGSADNERL
A. pleuropneumoniae DHAVVADSLLAKQVEILRGSSTLLYASSSPAGIVNVVDKRIPTEIPEKGYEVELNSRFDTAAKEKV
B. pertussis        DHAVTTEPLLADKIEVLRGPATLLYGGGAIGGVVNVLDRKIPTAVPQQGIEAEAELRGATGTKERA
                    ***: .:. *:. :*::**. :***...  .*:::: * :**        .     *  :.  *:

N. meningitidis     TSGGINIGLGKNFVLHTEGLYRKSGDYAVPRYR--------------------NLKRLPDSHADS
M. haemolytica      TYAGSTFALGNHLALRVQGMYNKASEYYAPHFTI-----------------EGKPYHRVPDSDVQS
A. baumanii         TTAGVTVGLSPRVALRAEGLYRNAGNYKTPHYQSSSYNSLEDLEN---QNIVYKNLKYLPESWAES
M. catarrhalis      ITASAEAPLGEHVAVRVAGLSKQAADYKTPRFDRHVFNKKHEDDNTQPEFIYKDTLKHLPDSHAKS
H. parasuis         YALGTTIGIGKHLALRLEGLDRQSQNYKVPQIKL-----------------GETLNYVPDTYHQS
P. mutocida         VNVALDVGLSKHFALHLEGLHKKAGDYRTPSYQY----------------QGSTHHKLANSFVDN
A. pleuropneumoniae GALGATFGIGKHIAVRAEGLTRHSDNYRVPGINL-----------------GERLNYVPDTYNKS
B. pertussis        GAIGITAGSG-NFAVRVEGLKRRSSDYRVPDWPD---------------------GKLAGSYSES
                       .     . ...::  *: ..: :* .*                            :. :  ..

N. meningitidis     QTGSIGLSWVGEKGFIGVAYSDRRDQYGLPAHSHEYDDCHADIIWQ--KSLINKRYLQLYPHLLTE
M. haemolytica      QTGTVSLSWIGERGHLGIAYTDRRDKYGLIGHTHKYDHYTISIIRQ--AVMFAKGYLRFYPHLAEE
A. baumanii         RLGTLGLSWIDDNTYLGVSYTHRHDEYGLPAHSHLYEGCGASAISINTRISGLKNYLLYYPQLMEE
M. catarrhalis      NAGTLGVSWVGNQGFLGASVSLRRDKYGLPNHSHEYEECSVHGISQ--SALQYKPYLRLYPFLMEN
H. parasuis         KVGTIGLSFIGEKGYLGASYNQRKDRYGLPGHNHKFDTCIAHIYDM--RLQGKHSYTNLYPHLMSD
P. mutocida         RSGSVGLSWVGDKGYLGVAYSQRKDKYGLPAHSHLYDEYYMHVLLS--DAHWRKPYLKHYPFLMEE
A. pleuropneumoniae KVGTLGLSFVGEQGYIGASYSKRRDNYGLPGHNHKFDFCIGHIYGN---KQGKYAYTYLYPHLIGE
B. pertussis        GQGTVGMSWITPRGYVGVAFTHLESKYGLPGHNHEYEGCHPHG----------------------
                      *::.:*::  . .:* : .  ...***  *.* :  :

N. meningitidis     EDIDYDNPGLSCGF-------H--------DDDNA--HAH-THS--GRPWIDLRNKRYELRAEWKQ
M. haemolytica      GDIDYNNPGIRLL------HTHIPGGSH------YGQDT-HEH---GKPWIDMHSKRYDIDGSLQN
A. baumanii         QDINYVNPRPDCH---------------QH--NHIHETT-FSH---NAPYIDLNTRRYDMRGEFTQ
M. catarrhalis      DDLEFDNAGLECHTHDD------HDHEHDHAHDHEHDHE-HDH---GKPWIDLKMKRYDVQGQINA
H. parasuis         EMVT-ENPHFHCGTD------YDLDPSHSHDHPYGHDHD-HTH---IGPWVDLHSKRIDIKGEIKQ
P. mutocida         TDIDYNNPGIDCIKKEWHSHGHLCNHGHAHHGNGQHSHDHHAH---ADPHIALNTQRWDLRGEWKN
A. pleuropneumoniae ENIG-SNPHFHCGTD------HAEDGTHSHDNPFGHDHD-HTH---PGPWVDLESKRFDVKAELRQ
B. pertussis        -------SHLHCGGHDD------HGHDEHEEGEAEHDHG-HEHGAGDVPYVKLRSNRTDLRAEYTD
                            .                                 *     * : :. .* :: ..

N. meningitidis     PFPGFEALRVHLNRNDYRHDEKAGDAV------------------ENFFNNQTQNARIELRHQ---
M. haemolytica      PLPGFEEAKISANYVDYYHDEKDGKRV------------------ENYFKNKGKNLRFELVHK---
A. baumanii         PFTGIDKIRTSLSYIDYFHNELEGDKI------------------TNFFKNTGKVGRIELSHQ---
M. catarrhalis      PFAGIDKIRASMGKVDYHHDEIDGGEK------------------TSFFDNQANVWRLEASHTPIH
H. parasuis         PLPMLDKIQLSYAQTDYYHDEKDAGKSGDTINPNRVDKSKDFGKPVNIFKNQGKNARLEFFHT---
P. mutocida         PVKGLDKVRFSIAKVGYRHDEKSGAIS------------------DNSFKNKGYSARVEFLHQ---
A. pleuropneumoniae PFKGIDKIKVSYADADYYHDEKDAGVLATRYH-KQLKKDQDYGKPVNIFKNRGKNARLEIYHA---
B. pertussis        PFAGFEKIRFRGGLTDYRHDEIEGGQL------------------GTRFQNRGYDARLELTHR---
                    *.  ::  :      .* *:*  .                      . *.*     *.*  *

N. meningitidis     -PIGRLKGSWGVQYLQQKSSALSAIS-------EAVKQPMLLDNKVQHYSFFGVEQANW-DNFTLE
M. haemolytica      -EWKGLKGAIGVQYTNQSTSALALEASRAA--KVFNKQPLLNNPKTKLWSLFAIERLNL-GDFTFE
A. baumanii         -PLGELTGILGLQYLEQDNSALSPVHSQEGHTTYLDTQQLLNRNVTKNFSVFGLEKYNW-NDFTFE
M. catarrhalis      TPMGKFSGVFGVGYLTSKNSGLVPPRYEDG--NKQDTQNILHNNKTKTGSVFWFEEYKPNDKLTVD
H. parasuis         -PIGGLTGMFGVQYQTLQSSANTPNN--------REVQWPLVDNRNKQISLFALEQYAW-DNFAIE
P. mutocida         -PIAGVSGLIGLSHVYQDSYALDNHTL------EYRKQNLLSDHTTAQQSLFLMEHVEL-GKWQFD
A. pleuropneumoniae -PLGGLTGVWGVQYQTQKSSMHAPKD--------REVKFPLVENTNKQMSLFGIEQYMW-DNFALE
B. pertussis        -PLYGWHGVVGVQTSYSDFRA-------------TGEEAFLPRSKTRAHGLFLLEEYRW-ADWRVE
                     *  *:     .                         :  *        ..* .*.     .  .:
```

```
N. meningitidis    GGVRVEKQKASIQYDKALIDRENYYNH----------------------PLPDLGAHRQTARSFAL
M. haemolytica     LSGRAERQKIAMDYDVKLIDRWLGFNT----------------------PMPNLDPHKDKGYSYSF
A. baumanii        LGARIEKQKVSMDYDIEKIKDSMKPWPNKYNSPYVEKNNKIRAQNLK-SILEAVQPNKETAFSYAG
M. catarrhalis     AAARIEKQTITMDYDKDAIYQSLNLGLATAHEPDI--------RFKRLLDSGTLNPKKQTARSYAV
H. parasuis        LGLRTEKQNIHIDYDLAKIQKQQKFNERT---------------YGK-QVDPDLSDYDEKAISYTG
P. mutocida        IGGRVEKQRIAMKYHFNVPKDEQP-------------------------PEELTRPHKSKAYSYAL
A. pleuropneumoniae FAGRVEKQKIEIEYDRNEIKRLQDHYRIS---------------GGK-QVEPDLSPYNQNAYAYSS
B. pertussis       LGARQDWQRVSPQ--------------------------------------GGAPASRTAGTSLSA
                   . * : *    .                                                . : :

N. meningitidis    SGNWYFTPQHKLSLTASHQERLPSTQELYAHGKHVATNTFEVGNKHLNKERSNNIELALGYEGDRW
M. haemolytica     ATHWYFAPNHKLTLNAAHQERLPNAQELYAHGKHIALNAFEAGNKNLKKERSNQIELSLAYVGDKW
A. baumanii        TVHWRFAPNYILSLTGTHQERLPNAQEMYTHGMHLATNSFEIGNRFLRKEKSNNLEISLAYKDDLL
M. catarrhalis     GTHLQLTPKHKLSLNLSHQERLPNAQELYAHGMHLATNSFEIGNRFLNKEKSNNIDLGLTFQGDKW
H. parasuis        AFNWFFHPDYQLSFTASHNERLPTPMELYYHGQHLATNSFEYGNKDLKKEISNNFELGLGYHTEKL
P. mutocida        SANYQLNEQHQFNMIVSHQERLPNAQELYAHGKHLATNSFEAGNKNLTKERSNNVELGWGYTGEKL
A. pleuropneumoniae TLNWFFHPDYQLSFTASHNERFPTPMELYYHGQHIATNSFEYGNKDLKKEQSNNVELGLGYQTERV
B. pertussis       AAIWDFAPQYSLALSVSRSQRLPSAQELYADGVHLATNTYEIGDPGLDRETSRNVDLTLRKHSGDT
                        :  .: : :  ::.:*:*.. *:* .* *:* *::* *:  * :* *.:.::

N. meningitidis    QYNLALYRNRFGNYIYAQTLNDGR---------GPKSIEDDSEMKLVRYNQSGADFYGAEGEIYFK
M. haemolytica     DYKLNLYHTRYGNYIYPLTLNDNR---------GPKSFTDEYNLKVNRYYQGEARFSGAEGEIGYL
A. baumanii        DYQISTYYYDFDNYIYLQTLNEVL---------GTTKVRDQHTLRINHYSQSAANFYGLEGNIGYQ
M. catarrhalis     DYRLGGYHYDFDNYVFLQTLSQYK--------QGLRGMRHDKDLKTARYEQAAAKFYGFDVNIGYQ
H. parasuis        DYKLSTYYNNFDNYIYNETLYR------------------SNNLFMRRYNQAKATFYGLEGIINYR
P. mutocida        GIKLSGYYQQFSNYIYAAILNNKTSCPWRPNSRCLRSLSDDYPLRLYRYNQAKAKIYGLEAEVSYQ
A. pleuropneumoniae GYKVNVYYNHFKNYIYNENLFR------------------ENQLFMRRYNQAKARFYGIEAEASYR
B. pertussis       TFSVSAFHNRVKNYIYANTLDR------------------YEDFRLIEYTQRDAEFTGVEGEIRHR
                     :  :     **::   *                         :   .* *  * : * :    .

N. meningitidis    PTPRYRIGVSGDYVRGRLKNLPSLPGREDAYGN------------RP------------FIAQDDQ
M. haemolytica     FTPNYRLAVFGDYVRGKLVNLPNIAMSYNIWTGE------------V----------DKWASQPDI
A. baumanii        FNSVYHGSLFGDYVKGRLTNLPDAVIAYDIWNRE-----------------------PTLAPQKDR
M. catarrhalis     INDVYHVALFGDYIRGKLTNLPDKKGRTDAYGN------------RP------------LIKQPDS
H. parasuis        FTPDYQFSVFGDMVKGKLKQLPDIKGLNDVYGEPILNPDYDPEYDEPEDQYYRPYLGKEMIKQADR
P. mutocida        ISSTHSVSIFGDYVRGKLKDLPSLPIGYKYIYNE----NYDMVGVQP----------TGWEKQPDG
A. pleuropneumoniae FNDKYQATIFGDMVRGWLTNLPPLTVNSDYSVFK----DYLPKDAKPG--------EDYLIYRADQ
B. pertussis       FGKVFSAAVFGDYVRGRLTG-------------------------------------------GGG
                       .   : ** ::* *                                             .

N. meningitidis    NAPRVPAARLGFHLKASLTDRIDANLDYYRVFAQNKLARYETRTPGHHMLNLGANYRRNTRYG--E
M. haemolytica     SAPRIPPLRLGARFNADFNLNWSGMLEYYRVFAQKKVSKYEQVTPGHHQVNLGVTYSNHFNQT--E
A. baumanii        YTPRLPPARLGTRLKADFDESLKGEIEYYRVFKQDNISKFEQVTSGYNMLNMTLAYKNKLSHT--E
M. catarrhalis     HTPRLPPKRLGMKLTANVNANWSGFLEYRHTFKQDKLANFERPTPAHNLVNLGLNYQHKPSHQAGS
H. parasuis        VSPRLPPIRLGARFNAQLTENLSGSVEWMKVFTQNKVSKLESSTKGYQLLNASLNYRRQIKGV--E
P. mutocida        NAPRMSPMRLGIKWNAYFDNGISFNTQLYRVFAQNKVARLETPTKGHTMLNLGMSYDGKMGNN--E
A. pleuropneumoniae NTPRTPPVRLGFRFNAEFTPNWSGDLELIRTFTQRRTSQLEYITEGNTMLNIGVAYSNKWKDL--D
B. pertussis       NLPRIPAARLGVRADAQWQ-NWAGGVEYFHVYRQDDIAAYESSTPGYDMVNATIRYRGKLDRT--A
                    ** .. *** :  *            :  :.: *   :  *  * .    :*    *  :

N. meningitidis    WNWYVKADNLLNQSVYAHSSFLSDT-PQMGRSFTGGVNVKF
M. haemolytica     YQVFLKVDNLLNQKMYQHASYLPHI-PQMGRNAMLGMNISF
A. baumanii        YDLFFKANNLLDQKVYAHETFLPYI-PQIGRNFSLGLNLNF
M. catarrhalis     VQVFFNANNLLNDKVFAHETFFPDM-PQMGRNFMLGANFKF
H. parasuis        YTVSLTGNNLLNQAVYIHNSYHPYV-PQMGRNFILGLDLSF
P. mutocida        YTLFANVNNVLNSRVYNHTSFLSYI-PQSGLGLNVGMNFKF
A. pleuropneumoniae YKISLNGTNLLNQPVYIHTSYHQFV-PQTGRNFILVVNVKF
B. pertussis       YEIYLRGNNLLNKLAFNHASFISTVAPLPGRSVLLGVRLTY
                           *:*:.  : * ::     *  * .      ..:
```

Figure S2. Alignment of ZnuD homologs. Aligned are the amino acid sequences of various pathogens. The histidine- and aspartic acid-rich stretches are highlighted in grey.
