# Supplementary material for: An Outer Membrane Receptor of Neisseria meningitidis Involved in Zinc Acquisition with Vaccine Potential
Source: PLoS Pathog. 2010 Jul 1;6(7):e1000969. doi: 10.1371/journal.ppat.1000969 (PMC2895646; doi:10.1371/journal.ppat.1000969)
Supplement: Table S1 — Primers used in this study. (0.05 MB DOC) [file ppat.1000969.s003.doc]

**Table S1.** Primers used in this study

| Name | Sequence | Relevant characteristics |
| --- | --- | --- |
| 0964-F | GAT**CATATG**CATGAAACTGAGCAATCGGTG- | NdeI site (bold) |
| 0964-R | GAT**GGATCC**TTAAATCTTCACGTTCACGCCGCC | BamHI site (bold) |
| Kan-R | TG**ACGCGT**CTCGACGCTGAGGTCTGC | MluI site (bold) |
| Kan-F | TG**TGTACA**GTCGACTTCAGACGGCCACG | NdeI site (bold) |
| ZnuD-F | GCAT**CATATG**GCACAAACTACACTCAAACCC | AatII site (bold) |
| ZnuD-R | AT**GACGTC**TTAAAACTTCACGTTCACGCCGCC |  |
| tonB-1 | GTACGATGATTGTGCCGACC |  |
| tonB-2 | ACTTTAAACTCC**GTCGAC**GCAA**GTCGAC**TGCGGGGGTTAA | AccI site (bold) |
| tonB-3 | TTAACCCCCGCA**GTCGAC**TTGC**GTCGAC**GGAGTTTAAAGT | AccI site (bold) |
| tonB-4 | GCCATACTGTTGCGGATTTGA |  |
| P1 | **GTCGAC**GGATCCGTGTAGGCTGGAGCTGCTTC | AccI site (bold) |
| P2 | **GTCGAC**GGATCCATGCCGTCTGAACATATGAATATCCTCCTTA | AccI site (bold) and DUS site underlined |
| zur-1 | TTCGCCGATGGCGGAATACA |  |
| zur-2 | CTTTCAGCGCAAA**GTCGAC**TCCGTCGACGCGTGCCTGTTC | AccI site (bold) |
| zur-3 | GAACAGGCACGC**GTCGAC**GGAGTCGACTTTGCGCTGAAAG | AccI site (bold) |
| zur-4 | TCCTATTGCGCAATACCCCC |  |
| znuA-1 | AAGGCGGGCTTTGGCACGTC |  |
| znuA-2 | TGTTGTGGCG**GTCGAC**TCCGGTCGACGAGGCGGTCAGCAA | AccI site (bold) |
| znuA-3 | TTGCTGACCGCCTC**GTCGAC**CGGAGTCGACCGCCACAACA | AccI site (bold) |
| znuA-4 | CAGGAGGAAATGTGGATGCCAA |  |
| znuAq1 | TCGGAGCCAACCAAGATACG | Used for qPCR |
| znuAq2 | CAAGTCCTAAGCCGTTGAGCA | Used for qPCR |
| znuDq1 | GCATCCACGCTTCGCAATA | Used for qPCR |
| znuDq2 | TTTCGCCGTGATGGTTCAA | Used for qPCR |
| rmpMq1 | CAGGCTCCGCAATATGTTGA | Used for qPCR |
| rmpMq2 | GTTGTCTTGAGCTTCGGCG | Used for qPCR |

All primers except those for the kanamycin- and chloramphenicol-resistance gene cassettes were designed based on the MC58 genome sequence.
